# Effect of background noise on neuronal coding of interaural level difference cues in rat inferior colliculus

**DOI:** 10.1111/ejn.12914

**Published:** 2015-05-06

**Authors:** Yasamin Mokri, Kate Worland, Mark Ford, Ramesh Rajan

**Affiliations:** 1 Department of Physiology, Monash UniversityMonash, Vic., 3800, Australia; 2 Ear Sciences Institute of AustraliaPerth, WA, Australia

**Keywords:** auditory midbrain, background noise, neural encoding, sound localization

## Abstract

Humans can accurately localize sounds even in unfavourable signal-to-noise conditions. To investigate the neural mechanisms underlying this, we studied the effect of background wide-band noise on neural sensitivity to variations in interaural level difference (ILD), the predominant cue for sound localization in azimuth for high-frequency sounds, at the characteristic frequency of cells in rat inferior colliculus (IC). Binaural noise at high levels generally resulted in suppression of responses (55.8%), but at lower levels resulted in enhancement (34.8%) as well as suppression (30.3%). When recording conditions permitted, we then examined if any binaural noise effects were related to selective noise effects at each of the two ears, which we interpreted in light of well-known differences in input type (excitation and inhibition) from each ear shaping particular forms of ILD sensitivity in the IC. At high signal-to-noise ratios (SNR), in most ILD functions (41%), the effect of background noise appeared to be due to effects on inputs from both ears, while for a large percentage (35.8%) appeared to be accounted for by effects on excitatory input. However, as SNR decreased, change in excitation became the dominant contributor to the change due to binaural background noise (63.6%). These novel findings shed light on the IC neural mechanisms for sound localization in the presence of continuous background noise. They also suggest that some effects of background noise on encoding of sound location reported to be emergent in upstream auditory areas can also be observed at the level of the midbrain.

## Introduction

Sound localization can be impaired by background noise, but it is often forgotten that we maintain relatively good sound localization performance across many signal-to-noise ratios (SNRs) (Good & Gilkey, 1996; Good *et al*., 1997; Lorenzi *et al*., 1999; Andéol *et al*., 2011; Kerber & Seeber, 2012). Some limited data are available on the neural bases of the effect of background noise on coding of cues for sound location or on the spatial tuning in auditory cortex and superior colliculus (SC) (Brugge *et al*., 1998; Furukawa & Middlebrooks, 2001; Martin *et al*., 2010). With respect to response strength, the effects in SC (Martin *et al*., 2010) and A1 (Brugge *et al*., 1998) seem to differ. In A1, continuous background noise only induced suppression of the responses similar to that seen simply by decreasing signal level, while in SC most responses were unaffected by noise, and only in a small number of cells did noise result in suppression or enhancement of the responses.

The inferior colliculus (IC) is an important obligatory midbrain auditory nucleus in relay of information from cochlea to cortex (Irvine, 1986; Pickles, 1988) and the source of indirect projections to SC (Slee & Young, 2013). It is also the site of *de novo* synthesis (Park, 1998; Pollak *et al*., 2003; Park *et al*., 2004; Li *et al*., 2010; Pollak, 2012) of sensitivity to interaural level difference (ILD) (Flammino & Clopton, 1975; Kelly *et al*., 1991; Oliver & Huerta, 1992; Palmer & Kuwada, 2005), the predominant binaural cue for azimuthal localization of high-frequency sounds (Erulkar, 1972; Mills, 1972; Irvine, 1986). Thus, investigating the effects of noise on ILD sensitivity in IC could elucidate if the different effects of noise on sound location coding in SC and A1 are emergent differences in these areas or whether common processes occur in IC, but are modified selectively in different upstream brain areas by local circuitry. Furthermore, IC neurons receive complex excitatory, inhibitory and modulatory inputs from several brain structures (Casseday *et al*., 2002; Pollak *et al*., 2002; Bajo & King, 2012), and it is plausible that selective effects of noise on these inputs can explain the mechanism underlying the changes in ILD sensitivity due to noise. However, the effect of noise on these inputs has not yet been assessed either in cortex or in the midbrain (SC or IC).

The aim of this study was to provide a better understanding of the effect of background noise on neuronal coding of ILD in IC of rats and to examine whether this effect can be explained by effects on the excitation and inhibition in this area. To do this, we first compared the ILD sensitivity functions in the absence and presence of binaural background noise, then compared the changes in ILD sensitivity functions as a result of introducing binaural vs. monaural background noise to assess the effect of noise on excitation and inhibition.

## Materials and methods

### Subjects

Recordings were performed in 25 adult male rats [ten Long-Evans and 15 Specific Pathogen Free (SPF) Sprague-Dawley], weighing 240–470 g, obtained from Monash Animal Services (Monash University, VIC, Australia). Experiments were approved by the Monash University Animal Experimentation Ethics Committee and conformed to the guidelines of the Australian Code of Practice for the Care and Use of Animals for Scientific Purposes.

### Surgical procedures

Animals were anaesthetized (60 mg/kg pentobarbitone, i.p.) and administered a muscle relaxant (0.1 mL xylazine-saline solution, i.p.). Anaesthesia was maintained with approximately hourly doses of 0.1 mL pentobarbitone and 0.05 mL xylazine. We routinely monitored depth of anaesthesia based on palpebral and pinch-withdrawal reflexes, and through continuous monitoring of the electrocardiogram (ECG) and electromyogram (EMG) from the forearm muscles. Body temperature was maintained at 37 ± 0.5 °C using a heating pad thermostatically controlled by feedback from a rectal probe.

An incision was made along the midline of the head and the overlying tissue was deflected to expose the skull. The auditory meatuses were transected close to the tympanic membrane with sufficient canal tissue left to form a seal around a sound delivery tube from the speaker coupler (see below), allowing sound to be delivered directly to the eardrum. A craniotomy was made in the skull over the left occipito-parietal cortex, extending 3 mm rostral from lambda and 4 mm lateral from midline, to insert the electrode for recording from the left IC.

### Recording procedures and confirmation of recording locations in IC

All recordings were done in a sound-proof room. The rat’s head was tilted 15° up from the horizontal, and the microelectrode was held in a stereotactic frame tilted 20° forward from the vertical. Parylene-coated tungsten microelectrodes with 2 MΩ impedance at 1 kHz (A-M Systems, Carlsborg, WA, USA) were used to record from the IC. The electrode penetration site on the cortex varied around positions approximately 1.1 mm rostral and 1.7 mm lateral of lambda. Signals from the microelectrode were amplified (gain = 1000) and band-pass filtered between 0.5 and 9 kHz. Spikes were extracted in real-time using a Schmitt trigger device with the trigger level set manually by the experimenter, while monitoring the electrode output and trigger level on an oscilloscope.

The electrode was advanced through the occipito-parietal cortex towards the midbrain while presenting auditory stimuli that were varied between noise bands and tonal stimuli until detection of robust auditory responses with characteristics of IC recordings (see below). Once we were assured that the response characteristics were indeed those expected from IC neurons, the electrode was advanced in 2- to 5-μm steps to obtain recordings from a well-isolated single neuron (SNR of at least five times background recording level).

Mean spike counts from a well-isolated single neuron were computed within the windows that were set to capture different response components of the neuron (i.e. onset, late and sustained components; see below) and were stored for further offline investigations. The spike waveforms were monitored continuously to ensure the single unit recording state was maintained and the same waveform was present for all recordings.

After data collection from a single well-isolated cell at one depth, the electrode was advanced at least 100 μm from the recording depth while monitoring the signal from the recorded cell on the oscilloscope screen to ensure it was progressively reduced in size and ultimately buried in noise, before attempting to obtain recordings from the next well-isolated cell. This process ensured that there was no overlap between data from successively recorded single neurons.

To ensure that all recordings were done in the IC, we used the tonotopic sequence of the characteristic frequency (CF; frequency of greatest sensitivity) of successive neurons and the characteristics of the neural responses. Thus, we only report recordings from penetrations in which we observed a systematic increase in CF with systematic movements further ventrally (Syka *et al*., 1981) from the first point where auditory drive was first obtained in each penetration. Also, we observed that almost all neurons in the recording area had spontaneous activity, and the very few neurons that did not were interspersed amongst spontaneously active neurons located more dorsally and more ventrally. Finally, the majority of neurons from which recordings were obtained had sustained activity and, again, the neurons that did not were interspersed amongst neurons with sustained activity located more dorsally and more ventrally. These three characteristics differentiate IC recordings from recordings in the SC under similar anaesthetic conditions (Wise & Irvine, 1983). SC cells do not show a systematic progression in CF in dorso-ventrally orientated penetrations, they show little to no spontaneous activity and they predominantly have onset-only responses. All these properties of SC recordings were also confirmed in rats in our other experiments not reported here. In a few cases, post-mortem histology with Nissl stains was conducted, and microscopy confirmed the location of our recordings in central nucleus of IC, but this histology is no longer available and cannot be presented in this report.

### Auditory stimuli

As detailed previously (Rajan, 2000, 2001, 2005), tones and noise stimuli to each ear were synthesized using a Tucker-Davis Technologies (TDT; Alachua, FL, USA) auditory processor at sampling rate of ∼200 kHz and passed through separate programmable attenuators, before feeding into one of two Sennheiser HD 535 speakers each in specially designed custom-made housing leading out to a sound delivery tube placed in one external auditory meatus (Rajan, 1995, 1998). Sound pressure level (SPL) was measured by presenting the sound stimulus into one end of a closed-cavity coupler through the speakers, while the output was recorded at the other end using a 0.25-inch condenser microphone (Type 4135; Brüel & Kjær, Nærum, Denmark) connected to a measuring amplifier (Type 2606; Brüel & Kjær). The frequency response curves of the speakers for frequencies ranging from 1 to 40 kHz were determined by measuring the output of the speakers using this set-up, while a Gaussian white noise stimulus was played at the maximum voltage (±10 V for the TDT system), and the power spectral density of the recorded output was then obtained. This was adjusted by spot checks of output levels of tones at five frequencies across this range. The frequency response curves of the speakers resulting from this calibration were used for setting of the level of the sound stimuli (Rajan *et al*., 1991).

Tonal stimuli were gated tone bursts shaped with a 4-ms rise–fall time, with variable duration between 50 and 200 ms depending on the cells’ response profile (see below). Tonal stimuli were always at the CF. To test the effects of background noise, band-passed Gaussian white noise (1–40 kHz) was generated using the TDT system and was presented, either monaurally or binaurally, as a continuous stimulus starting 5 s before commencing testing with the tones and remained on for the duration of each block of testing (see below).

### Segregation of different temporal components of neural responses

Once a well-isolated single neuron was identified, we estimated the cell’s CF and the threshold at CF by monitoring the neural responses audio-visually while manually varying tonal frequency and level. Then, CF tone stimuli were presented at levels from CF threshold to 60 dB > CF threshold, to categorize the cell’s response patterns as *Onset*, *Onset-late* and *Sustained* (Irvine & Gago, 1990), for setting of stimulus duration for all subsequent tests. Typically, Onset responses showed a brief burst of responses (5–30 ms) shortly after stimulus onset (i.e. onset response component), Onset-late responses showed the onset response component, followed by a brief pause of at least 20 ms and then sustained firing for the duration of the stimulus (i.e. late response component), and Sustained responses had a sustained discharge for the duration of the stimulus, commencing about 35–50 ms after stimulus onset that was maintained while testing with a range of stimulus durations from 50 to 300 ms (i.e. sustained response component). During our recordings, onset response components were tested with 50-ms tones, and late or sustained response components were tested with tones ranging in duration for 100, 150 or 200 ms, with tone duration set to ensure that all response components were captured. Onset, late and sustained response components were captured separately with appropriate time windows set for data collection. Using different durations for tonal stimuli helped in saving time, given the large number of conditions tested for each cell in this study.

It has been shown that the onset and late components of Onset-late cells can behave differently in terms of absolute sensitivity at CF, patterns of growth of response rate with level and ILD sensitivity (Hind *et al*., 1963; Geisler & Rhode, 1969; Moore & Irvine, 1981; Irvine, 1986; Irvine & Gago, 1990; Finlayson, 1999). Of particular relevance to this study, the interaction between monaural inputs to a cell in the IC of rats can also be time-dependent (Zhang & Kelly, 2009, 2010). This was also true of our recordings, such that the effects of noise could be different on the two temporal response components even when recorded from the same IC cell (see Results). Hence, we separately analysed onset and late components of Onset-late responding cells and will present data to justify this segregation in the Results. These components, i.e. onset, late and sustained response, are referred to as *response components* hereafter.

### Quantitative data collection

As described earlier, the CF and the threshold at CF were first estimated by the experimenter for each cell. The first set of quantitative measurements was of the frequency-level response area to accurately confirm CF and threshold at CF for each response component. The frequency-level response area was determined with tonal stimuli presented over a wide frequency range encompassing the estimated CF and from levels extending from 20 dB below the estimated CF threshold up to 80 dB SPL, and with spike counts measured across 8–20 repetitions of this matrix of frequency-level combinations. The spike count at each frequency-level combination also allowed generation of input–output (I-O) functions of the relationship between neural response and tone sound level.

After determining the CF and CF threshold for each response component, and recording the frequency-level response area, we then examined sound location encoding characteristics of each response component by recording responses to variations in ILD. We used the average binaural level (ABL)-constant method (Irvine, 1986), in which ILDs were created by varying the sound levels in the two ears symmetrically around a pre-specified base level (ABL). ILDs were generated over a range from −30 dB (sound level in the ear contralateral to the recording IC set 15 dB > ABL and sound level in the ipsilateral ear set 15 dB < ABL) to +30 dB (sound level in contralateral ear set 15 dB < ABL and sound level in ipsilateral ear set 15 dB > ABL), in steps of 5 dB. At each ILD, the stimulus was repeated 20 times, and in each block, ILD stimuli were alternated so that a larger contralateral level was followed by a larger ipsilateral level to reduce any effect of adaptation. Spike count across these 20 repetitions was considered as the neural response to the tonal stimulus at that ILD.

For each response component, the spike count vs. ILD function, henceforth referred to as the ILD sensitivity function or ILD function, was first obtained at a particular ABL that corresponded to a contralateral sound level that when presented monaurally evoked robust but not saturated responses. This level was determined from the CF I-O function obtained from the frequency-level response area measurement and is referred to as the *standard ABL* hereafter. Then, the effect of binaural background noise on responses to variations in ILDs was examined. Noise level was initially set to the SPL that evoked a noticeable effect on but not total suppression of the ILD function. This noise level is hereafter referred to as the *standard noise level*.

After recording responses to ILD variation about the standard ABL in the presence of the standard binaural background noise, if recording was still stable, we recorded responses to ILD variation about the standard ABL in the presence of the standard background noise, but presented only to one ear at a time. In such tests, we also assessed the binaural interactions potentially underlying the ILD sensitivity function, categorizing cells based on their binaural input characteristics into EE, EI, IE, predominantly binaural (PB), and EO/mon, using the notation introduced by Goldberg & Brown (1969), and based on characteristics described in detail by Irvine *et al*. (1996). In this categorization, EI cells are classed as receiving predominantly excitatory input from the contralateral ear and predominantly inhibitory input from the ipsilateral ear, the reverse is true for IE cells, EE cells receive excitatory inputs from both ears, PB cells show strong binaural facilitation regardless of their monaural response characteristics (e.g. the EE/F cells among this group receive sub-threshold excitatory inputs from each ear, but when both ears are stimulated simultaneously, the response to the stimulus is much stronger than the summation of the responses to monaural stimulation) and EO/mon cells are monaural cells. In practice, in the EO/mon category, we found only cells receiving excitatory input from the contralateral ear. This classification would be used to speculate on the mechanisms underlying the effect of binaural background noise on ILD sensitivity based on the excitatory and inhibitory inputs to each response component, as are described in detail later.

Then, if recording conditions permitted, the effect of other background noise levels was tested on ILD sensitivity at the standard ABL. Finally, where possible, we examined other ABLs to investigate the effect of varying SNR (SNR = CF tone SPL – background noise SPL) on ILD functions.

### Classification of ILD functions

The main focus of this study was to assess the effect of background noise on ILD sensitivity of the IC neurons. As this effect may differ between neurons coding for different azimuthal locations, we grouped the recorded ILD sensitivity functions into six categories based on their putative functional role in the coding of sound location, using descriptors that have been identified and used in previous studies in the IC and other auditory structures to define ILD sensitivity (Irvine, 1986; Irvine *et al*., 1996; Uragun & Rajan, 2013).

**Figure 1:**
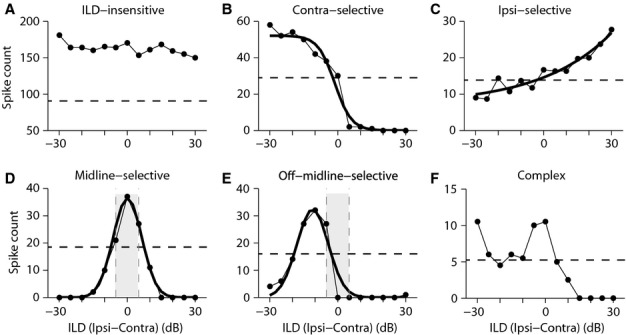
Examples of the different categories of ILD sensitivity functions in the absence of background noise. An example (A) ILD-insensitive (variations in response strength of <50% of the maximum response of the ILD function); (B) Contra-selective (monotonic); (C) Ipsi-selective (monotonic); (D) Midline-selective (non-monotonic); (E) Off-midline-selective (non-monotonic); and (F) Complex (multiple peaks) ILD function. Dashed horizontal line represents 50% of the maximum response margin [ILD function was ILD-sensitive if responses dropped below this level at some ILDs (B–F)]. Thick solid line shows the best fitting function (fit with the smallest SSE among Boltzmann, exponential, Gaussian and Lorentzian functions) to the ILD sensitivity function. This fitted function was used to categorize the ILD sensitive functions into categories shown in B–E and to compute some metrics characterizing the ILD functions, i.e. maximum response strength, ILD_50_, peak ILD, 80% dynamic range and width. It was not possible to find a good fit among the above mentioned functions for Complex ILD functions (F). The shaded area indicates the ILDs ranging between 5 and −5 dB [for Midline-selective ILD functions (D), peak ILD fell within this range, while for Off-midline-selective functions (E), it fell outside this range]. Ipsi: sound pressure level in the ipsilateral ear; Contra: sound pressure level in the contralateral ear.

For this classification, we first identified responses that were ILD-sensitive, defined as functions showing ILD-dependent variations in response strength of more than 50% of the maximum response of the ILD function, a well-established criterion for quantifying ILD sensitivity (Rose *et al*., 1963; Irvine *et al*., 1996). ILD functions that did not meet this criterion were classed as *ILD-insensitive* (Fig.1A; all ILD responses are larger than 50% of the maximum response, marked by the dashed horizontal line). We then categorized the ILD-sensitive responses into monotonic and non-monotonic ILD functions, based on which function among Boltzmann, exponential, Gaussian and Lorentzian functions (defined below) was a better fit to the ILD function. We used the *fit* function of the MATLAB *curve fitting toolbox, and* the best fit for each individual ILD function was defined as the fit with the smallest sum of squared errors (SSE). Determining the best fit for the ILD functions was particularly useful, as we used the parameters of the best fitting function for each ILD function for quantifying the characteristics of that ILD function, i.e. response strength, ILD_50_ or peak ILD, and full width at half maximum or 80% dynamic range (these characteristics are described later in detail).

A Boltzmann function is defined in eqn 1, where *A*_1_ is the initial activity, *A*_2_ is the final activity, *x*_0_ is the half amplitude point and *d*_*x*_ is descriptive of slope:

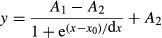
1

An exponential function is defined in eqn 2, in which *A*_1_ is maximum response, and *b* is the growth rate:

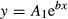
2

A Gaussian function is defined in eqn 3, in which *A*_1_ is the maximum response, *w* is indicative of width, *x*_*c*_ is the location of the peak response and *A*_2_ is the baseline activity:

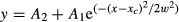
3

A Lorentzian function is defined in eqn 4, in which *A*_1_ is the maximum response, *x*_*c*_ is the location of the maximum response and *w* is width.

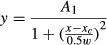
4

In all equations, *x* is the ILD at which the stimulus was presented, and *y* is the response to the stimulus at that ILD. Note that by using the inverse function of these functions, it is possible to determine the ILD (i.e. *x* value) at which a particular response strength, for example 50% of the maximum response (i.e. *y* value), occurred. We used this method to determine some of the characteristics of the ILD functions, such as ILD_50_, as will be described later.

If the best fitting function for an ILD function was either a Boltzmann or an exponential function, the ILD function was termed *monotonic*. Monotonic ILD functions were further divided into *Contra-selective* (Fig.1B) if the maximum response occurred at the ILDs favouring the contralateral ear [*A*_1_ < *A*_2_ in eqn 1 or *b *<* *0 in eqn 2], and *Ipsi-selective* (Fig.1C) if the maximum response occurred at the ILDs favouring the ipsilateral ear [*A*_1_ > *A*_2_ in eqn 1 or *b *>* *0 in eqn 2].

If the best fit was either a Gaussian or a Lorentzian function, and the response dropped by more than 50% of the peak response at both sides of the peak response within the range of ILDs tested, the ILD function was termed *non-monotonic*. Non-monotonic ILD functions were classed into *Midline-selective* (Fig.1D) and *Off-midline-selective* (Fig.1E) functions, based on whether the location of the peak response [*x*_*c*_ in eqns 3 and 4] was in the range of ±5 dB about 0 dB ILD (Midline-selective) or outside this range (Off-midline-selective). However, for some functions, although a Gaussian or Lorentzian function was the best fit, the response did not drop below 50% of the peak response at one side of the peak response, within the range of ILDs tested, and we classified these functions among the monotonic functions.

It was impossible to find a good fit for a few ILD functions with multiple peaks, and these ILD functions were categorized as *Complex* (Fig.1F; one maximum occurred at ILD −30 dB and one around ILD 0 dB). The SSE for the best fit for these Complex ILD functions constituted the outliers of the distribution of SSEs for the best fit for all ILD functions. Most Complex functions were similar to those reported earlier as *Trough* functions in A1 of cats (Irvine *et al*., 1996).

Although despite a long history at all levels of the auditory system of describing ILD functions as being monotonic (sigmoidal-shaped) or non-monotonic (peaked), there is no strong theoretical basis to guide what form of functions should be used to fit to ILD sensitivity functions in IC or other areas in the auditory pathway (Uragun & Rajan, 2013). Therefore, to determine if an ILD function was monotonic or non-monotonic, initially we used a sigmoidal function (Boltzmann) and one bell-shaped function (Gaussian). However, when we implemented this strategy, we found that these two functions did not capture the variability in the shape of the ILD functions. For example, we found that none of these initial fitting functions (Boltzmann or Gaussian) was a good fit for ILD functions like those shown in Fig.1C. Hence, and to have a more accurate estimate of the metrics characterizing the ILD functions, i.e. maximum response, ILD_50_, peak ILD, 80% dynamic range and width, we added two more functions that were a better fit to some ILD functions than were the Boltzmann or Gaussian functions, namely exponential and Lorentzian functions.

### Defining the features of the ILD functions

To characterize the effect of noise, we extracted some metrics to describe the main features of the ILD functions from the best fit found using the curve fitting method described above.

*Maximum response strength* at the preferred location: defined as the maximum of the ILD function. This value was equal to A_1_ + A_2_ in eqns 1 and 3, *A*_1_ in eqn 4, and the maxima for eqn 2. Equations 1 and 2 were for monotonic ILD functions and eqns 3 and 4 were for non-monotonic ILD functions.

*Putative coding features* of the ILD functions: defined as the ILD_50_ (for monotonic functions) or peak ILD (for non-monotonic functions). The ILD_50_ was defined as the ILD at which the strength of the responses was 50% of the maximum response. This is the inflection point at which the inputs from the contralateral ear dominate the inputs from the ipsilateral ear and is thought to have a role in population coding of sound location in IC (Palmer & Kuwada, 2005). The ILD_50_ was found using the inverse function (described earlier) of the best fitting function [eqns 1 or 2]. For non-monotonic functions, peak ILD signals the preferred location of a cell and was defined as the ILD at which the peak response occurred [*x*_*c*_ in eqns 3 and 4].

*Tuning characteristics* of the ILD functions: defined as 80% dynamic range for monotonic ILD functions and width for non-monotonic ILD functions. The 80% dynamic range was the ILD range over which response strength dropped from 90 to 10% of the maximum response strength. ILDs at which the 90 and 10% of the maximum response occurred were found using the inverse function of the fitted functions, i.e. eqns 1 and 2. Width for non-monotonic functions was taken as the width of the ILD function at the response strength that was half the maximum response, i.e. full width at half maximum (FWHM). This was equal to 2.35*w* in eqn 3, i.e. full width at half maximum for a Gaussian function, and *w* in eqn 4.

## Results

In this section, we first categorize responses based on their putative functional role in encoding location of a sound source, and then show how introducing binaural background noise affected this spatial coding. Finally, we describe the potential correlation between the effect of background noise on the excitatory and inhibitory inputs received from individual ears and the effect of binaural background noise on spatial coding.

### IC cell response patterns

In total, we recorded from 67 cells, confirmed by their response characteristics and the progression of CF in successively encountered neurons to be in the central nucleus of IC (see Materials and methods). We classified cells as Onset (47.7%), Onset-late (40.2%) and Sustained (11.9%) using the time pattern characteristics of responses to CF stimuli (Irvine & Gago, 1990). This distribution was similar to that reported for the cat IC (Irvine & Gago, 1990). In most cells, the response pattern remained constant with CF tone level tested over a range from threshold to 60 dB > CF threshold. In the few cells where there were changes, we found that from levels of 20 dB > CF threshold (used in our study for these cells) there were no changes in the response pattern of an individual cell.

Similar to other studies mentioned earlier, it was apparent in our recordings too that the different time components of the same Onset-late cell’s response could show very different ILD sensitivity, and we also found that even when they showed the same form of ILD sensitivity, the effects of noise could be quite different for different time components of the same cell (see below). Therefore, we separately analysed the onset and late components of Onset-late responding cells, resulting in a total of 94 response components for analysis. These components could be easily separated temporally, because they were separated by a gap of at least 20 ms.

### Classification of responses based on ILD sensitivity at the standard ABL

As described in detail in the Materials and methods, to examine ILD sensitivity, a tonal stimulus at the CF of each response component was presented at ILDs varying around the *standard ABL*. Spike counts recorded in response to the tonal stimulus at each ILD constituted the ILD sensitivity function (ILD function) for each response component.

**Figure 2:**
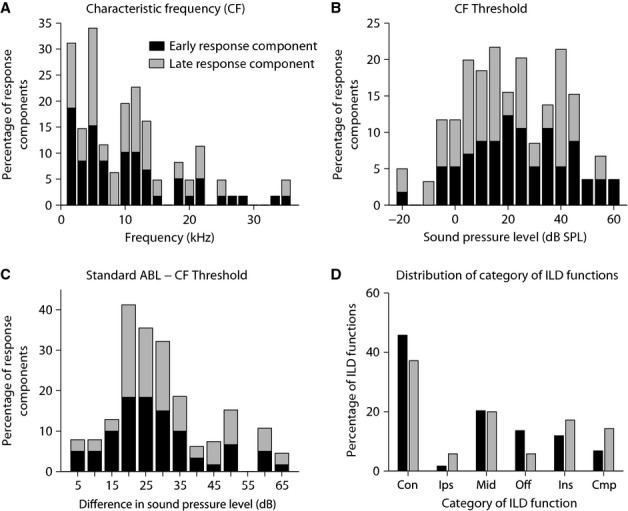
Characteristics of the neuronal population examined (94 response components) separated for early (onset) and late (late and sustained) response components. Distribution of the (A) characteristic frequency (CF; frequency of greatest sensitivity); (B) CF threshold; and (C) difference of standard ABL and CF threshold (standard ABL = ABL equal to the contralateral sound level that evoked robust but not saturated responses). For each response component, i.e. onset, late or sustained component of a cell’s response, the ILD function was first obtained at the standard ABL of that response component. (D) Distribution of categories of ILD functions. Con: Contra-selective; Ips: Ipsi-selective; Mid: Midline-selective; Off: Off-midline-selective; Ins: ILD-insensitive; Cmp: Complex.

Figure2 shows the distribution of the characteristics of the test population (namely CF, CF threshold, and the difference between the standard ABL and CF threshold) separated for early and late response components. The CF for the 94 response components ranged from 0.8 to 34 kHz (Fig.2A), with most response components having a CF < 15 kHz, and the CF threshold ranged from −20 to 60 dB SPL (Fig.2B), with most CF thresholds lying between 5 and 40 dB SPL. There was no significant difference between the distribution of CF or CF thresholds for the two response components (two-sample Kolmogorov–Smirnov test, *P* = 0.5 and *P* = 0.1 for CF and CF threshold, respectively). However, note that while in our recordings the CF was the same for the early and late response components of the same On-late cell, for seven out of 27 On-late cells, the CF threshold for the early response component was different from the CF threshold of the late response component of the same cell. As shown in Fig.2C, for most response components (70.5%), the standard ABLs at which ILD functions were recorded were 15–35 dB > CF threshold. Again, there was no significant difference between the distributions of this measure for early and late response components (two-sample Kolmogorov–Smirnov test, *P* = 0.8).

We divided the ILD functions into six categories: Contra-selective, Ipsi-selective, Midline-selective, Off-midline-selective, ILD-insensitive or Complex, based on the criteria described in the Materials and methods. Figure2D shows the distribution of the categories of ILD functions separately for early and late components of the responses (early responses in this figure are the onset response components of the Onset and Onset-late responses, and late responses constitute the late response components of Onset-late responses and the sustained response components). In general, the distributions of ILD function categories were quite similar across early and late responses (two-sample Kolmogorov–Smirnov test, *P* = 0.8). For both temporal response components, the major class consisted of Contra-selective cells, with non-monotonic functions (Midline-selective and Off-midline-selective functions) being the second largest category. ILD-insensitive and Complex ILD functions formed the next largest categories, with slightly more of the late response components showing these types of ILD functions than the early response components.

**Figure 3:**
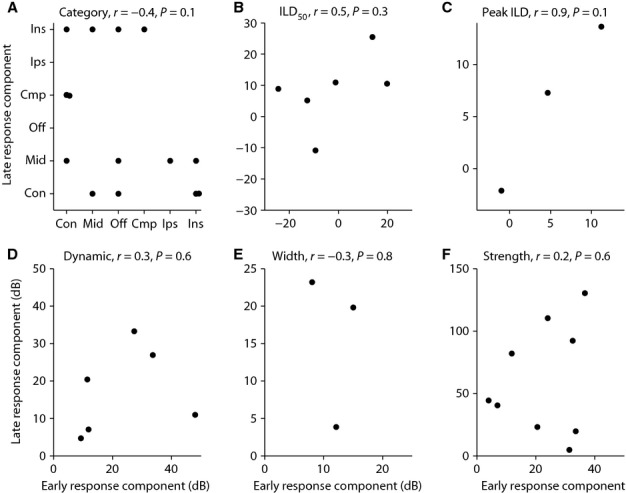
Comparison between the ILD sensitivity of the onset and late response components of the Onset-late cells. (A) Comparison between the category of the ILD function in the absence of background noise for the onset and late response components of the same onset-late cell when the categories of the ILD functions for these response components for the same cell were different from each other (14 cells). Comparison between the (B) ILD_50_; (C) Peak ILD; (D) 80% dynamic range; (E) width; and (F) maximum response strength of the ILD functions of the onset and late response components of the same onset-late cell when the categories of the ILD functions for these response components for the same cell were the same. ILD_50_ and 80% dynamic range were used to characterize Contra-selective and Ipsi-selective (monotonic) ILD functions (six cells), while Midline-selective and Off-midline-selective (non-monotonic) ILD functions (three cells) were characterized by peak ILD and width. Maximum response strength was compared for all the cells with monotonic or non-monotonic ILD functions (nine cells). There was no correlation between these quantities, as can be deduced from the *r* and *P* values reported for each metric. Due to these differences between the ILD functions of the onset and late response components of the same onset-late cell, among other reasons, we analysed these response components separately. Con: Contra-selective; Ips: Ipsi-selective; Mid: Midline-selective; Off: Off-midline-selective; Ins: ILD-insensitive; Cmp: Complex; *r*: correlation coefficient; *P*: *P*-value for testing the hypothesis of no correlation.

Despite this overall similarity of distributions, considering the Onset-late cells, onset and late response components of an individual cell did not routinely show the same ILD sensitivity category. For 14 out of 27 Onset-late cells, the category of the ILD functions obtained was different for onset and late response components, in agreement with other studies that have also reported differences in the ILD sensitivity of early and late responses (Hind *et al*., 1963; Geisler & Rhode, 1969; Moore & Irvine, 1981; Irvine, 1986; Irvine & Gago, 1990). Figure3A plots, for these 14 Onset-late cells, the category of ILD function of the onset response component vs. that for the late response component of the same cell, and shows that there was no relationship between the ILD function categories of the two components. For example, when the category of the ILD function for the onset response component was Contra-selective, the category of the late response component could be Midline-selective, Complex or ILD-insensitive.

The remaining 13 Onset-late cells each had the same category of ILD functions for the onset and late response components. For nine of these cells, the ILD functions of their onset and late response components were either both monotonic or both non-monotonic ILD functions, which enabled us to compute the metrics characterizing the ILD functions for these functions. Figure3B–F show the comparison between these metrics for the onset and late response components of these nine Onset-late cells (for the remaining four Onset-late cells, both onset and late response components were ILD-insensitive and it was not possible to compute these metrics for comparison). These metrics were the ILD_50_ for monotonic functions (Fig.3B), peak ILD for non-monotonic functions (Fig.3C), 80% dynamic range for monotonic functions (Fig.3D), FWHM for non-monotonic functions (Fig.3E) and maximum response strength (Fig.3F). As shown by the *r* values at the top of each panel, there was no significant correlation between the metrics characterizing the ILD functions of the onset and late response components of these Onset-late cells. Even though the *r* value for the peak ILD for the three non-monotonic ILD functions (Midline-selective and Off-midline-selective) was high, the relationship was not significant (*P* = 0.1). Thus, even in cells with the same ILD function category for onset and late response components, there was rarely any correlation between the metrics for the ILD functions for the two response components. These differences formed the basis for treating the early and late response components of the Onset-late cells as separate, and analysing the effect of background noise on these components separately.

### Effect of binaural background noise on coding of ILDs at the standard SNR

We now detail the effect of introducing binaural background noise on ILD functions recorded at the standard ABL and standard background noise level, i.e. at the standard SNR.

For seven out of 94 ILD functions, none of the noise levels tested had a strong effect on the responses, so these ILD functions were discarded from further analysis, leaving 87 ILD functions for analysis at the standard SNR. Of these 87 ILD functions, 13 were ILD-insensitive and eight were Complex, and these are considered separately later. Effects described here are therefore for 66 ILD functions (38 Contra-selective, three Ipsi-selective, 17 Midline-selective and eight Off-midline-selective ILD functions). For these functions noise could be causing the following.

#### Change in maximum response strength

**Figure 4:**
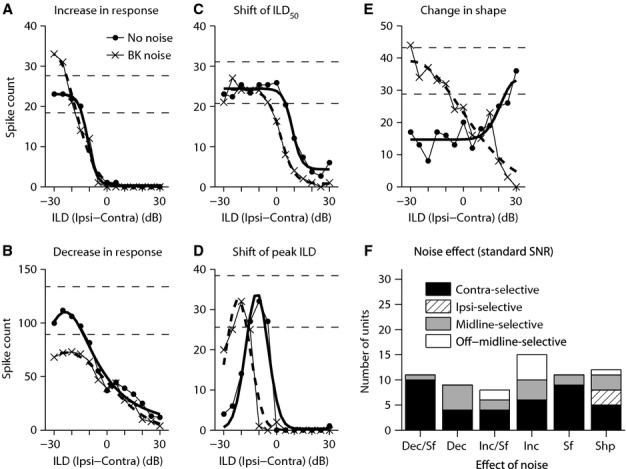
Different categories of noise-induced changes to the ILD functions. Noise induced (A) an increase in maximum response strength; (B) a decrease in maximum response strength; (C) a shift in ILD_50_ for monotonic ILD functions – in this example, ILD_50_ was shifted towards ILDs favouring more contralateral locations in the presence of background noise (‘BK noise’ ILD function) compared with the no-noise condition (‘No noise’ ILD function); (D) a shift in peak ILD for non-monotonic ILD functions – in this example, the peak ILD was shifted towards ILDs favouring the contralateral ear in the noise condition (‘BK noise’ ILD function) compared with the no-noise condition (‘No noise’ ILD function); (E) a change in the shape (category) of the ILD function – in this example, the category of the ILD function changed from Ipsi-selective (‘No noise’ ILD function) to Contra-selective (‘BK noise’ ILD function). In A–E, the thick solid curve is the best fit to the ILD function in the no-noise condition and the thick dashed curve is the best fit to the ILD function in the noisy condition. These best fits were used to compute the metrics required for classifying the effect of noise (A–E), i.e. maximum response strength, ILD_50_, peak ILD and category (shape) of ILD function. The horizontal dashed lines represent the 20% margin from the maximum response in the no-noise condition. If the maximum response strength of the ILD function in the noise condition was outside this range, the change in maximum response strength compared with the no-noise condition was considered significant (note the maximum of the ‘BK noise’ ILD function in A and B). (F) Distribution of the effects of binaural background noise on the different categories of ILD functions for all ILD functions tested at the standard SNR (66 ILD functions). At the standard SNR, the ILD function was obtained at the standard ABL, and background noise was at the standard noise level, i.e. noise level that evoked a noticeable effect on the ILD function but did not completely suppress the responses. The standard SNR was the highest SNR tested for each ILD function within the noisy conditions. Dec: decrease in response strength; Inc: increase in response strength; Sf: shift in ILD_50_ for monotonic ILD functions or peak ILD for non-monotonic ILD functions; Shp: change in the shape (category) of the ILD function; Dec/Sf: decrease in response strength accompanied by lateral shift of ILD_50_ or peak ILD; Inc/Sf: increase in response strength accompanied by lateral shift of ILD_50_ or peak ILD; SNR: signal-to-noise ratio (=CF tone sound pressure level – background noise sound pressure level); Ipsi: sound pressure level in the ipsilateral ear; Contra: sound pressure level in the contralateral ear.

The noise-induced change in maximum response strength was considered significant if the maximum response in the presence of noise differed from that in the absence of noise by 20% of the maximum response strength in the latter condition. The value of 20% was chosen because it appeared to demarcate changes in maximum response strength due to noise masking from changes explicable as random variability, and also because it is the criterion used by Zhang & Kelly (2010) for similar quantification of changes in responses. We tested some values other than 20% as well and found that small changes in this value did not affect the overall results significantly. Figure4A shows an example ILD function that exhibited a noise-induced increase in maximum response strength by 42.8% of the maximum response strength in the no-noise condition, while Fig.4B shows an example ILD function that exhibited a noise-induced decrease in maximum response strength by 35.4% of the maximum response strength in the no-noise condition.

#### Shift of ILD function

Noise could also cause a shift in the ILD function towards more negative or more positive ILDs (i.e. to ILDs favouring the contralateral or ipsilateral ear, respectively). For monotonic ILD sensitivity functions, this change was quantified by comparing the ILD_50_ values across noise conditions, while for non-monotonic ILD sensitivity functions, this was done by comparing the peak ILDs. A shift of more than 5 dB or <−5 dB in these parameters was considered significant. Figure4C shows an exemplar monotonic ILD function for which the ILD_50_ shifted by 6 dB towards more contralateral locations, compared with the ILD_50_ of the ILD function in the no-noise condition, when background noise was introduced. For the exemplar non-monotonic ILD function in Fig.4D, the peak ILD in the presence of noise was shifted by 10 dB towards more contralateral locations, compared with the no-noise condition.

Background noise could result in a combination of these changes in an ILD function, e.g. a significant decrease in response strength accompanied by shift of the ILD function to more lateral ILDs.

#### Change in category

Introduction of background noise sometimes resulted in a change in the shape (i.e. the category) of the ILD function, as shown for the example ILD function in Fig.4E.

#### Change in tuning characteristics

Tuning of the ILD functions was measured for non-monotonic functions as the width of the ILD function and for monotonic ILD functions as the 80% dynamic range, and introducing background could affect these metrics. Change in these features of ILD functions was not used to classify the effect of background noise, and it is reported only for completeness of description.

We used changes in maximum response strength, putative coding features, i.e. ILD_50_ and peak ILD, as well as in the shape of the ILD function to classify the effects of the standard level of background noise on the ILD sensitivity function at the standard ABL, and these effects of background noise on the 66 ILD functions available for analysis are summarized in Fig.4F. With respect to response strength, almost equal numbers of ILD functions exhibited suppression in maximum response strength (30.3%) (Fig.4F; ‘Dec’ and ‘Dec/Sf’), as exhibited enhancement of maximum response strength (34.8%), (Fig.4F; ‘Inc’ and ‘Inc/Sf’). One interesting result, with respect to effects in the different ILD function categories, was that for nearly all Off-midline-selective functions (seven/eight), binaural noise increased the maximum response strength (the clear bars marked as ‘Inc’ and ‘Inc/Sf’ in Fig.4F), in contrast to Midline-selective functions where noise also decreased the maximum response strength. For many other ILD functions (16.6%), while maximum response strength was maintained, there was a shift in ILD_50_ or peak ILD (Fig.4F; ‘Sf’). Shift in ILD_50_ without or with changes in maximum response strength occurred in 56.1% of monotonic functions, while shift in peak ILD occurred in 28% of the non-monotonic functions (Fig.4F; ‘Sf’, shift only; ‘Dec/Sf’, decreased response strength + shift; ‘Inc/Sf’, increased response strength + shift). This suggests that the peak ILD of non-monotonic functions may be more resilient to noise compared with the ILD_50_ of monotonic functions. The shift of the ILD functions was always towards ILDs favouring more contralateral locations compared with the no-noise condition except for two Contra-selective functions and one Mid-line-selective function, for which the shift was towards more ipsilateral locations. The monotonic ILD functions discussed here were all Contra-selective, as the only effect observed for Ipsi-selective functions was that background noise altered the shape of the ILD function (note hatched bars in Fig.4F only at label ‘Shp’). Finally, with respect to changes in tuning characteristics (not illustrated in Fig.4F for clarity), most non-monotonic ILD functions (61.1%) showed a decrease in tuning width, while there was an increase in the tuning dynamic range for most monotonic ILD functions (64.2%).

The effect of background noise was also studied for 13 ILD-insensitive and eight Complex ILD functions, which were not detailed above, as it was not possible to use the metrics described earlier to quantify the effects of noise on these ILD function categories. Most Complex functions (92.3%) exhibited a change in shape in background noise, while the rest showed an overall decrease in response across all ILDs. Twelve out of 13 ILD-insensitive functions turned into Contra-selective ILD functions in background noise. Only one of these ILD-insensitive functions exhibited an overall suppression in the responses and remained ILD-insensitive when background noise was presented.

For 13 out of 27 Onset-late cells, the category of the ILD functions for early and late response components was the same. However, for six out of these 13 cells (46.1%), background noise at the standard SNR produced different effects on onset and late response components. For example, for one of these cells, the effect of introducing standard background noise on the ILD functions of the onset response component was an increase in response strength, while it was a decrease in response strength for the ILD function of the late response component.

### Effect of variation of the level of binaural background noise on ILD sensitivity

**Figure 5:**
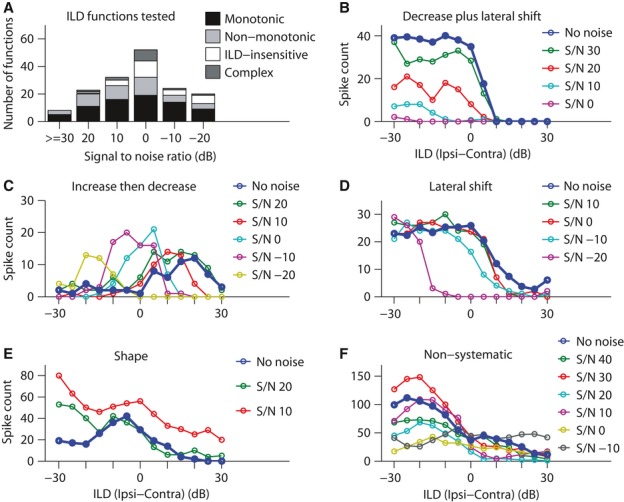
Different patterns of change in ILD functions at the standard ABL as level of background noise varied from standard noise level (standard SNR) to higher noise levels (lower SNRs). (A) Distribution of the number of ILD functions tested at each SNR (for 52 ILD functions). (B–F) When ILDs were tested at multiple SNRs, the patterns of change observed as SNR decreased were (B) decrease in maximum response strength, accompanied by shift of ILD_50_ or peak ILD – for the Contra-selective ILD function in this panel, as SNR decreased, the ILD_50_ shifted to ILDs favouring more contralateral locations compared with conditions with higher SNRs (including the no-noise condition); (C) increase followed by decrease in maximum response strength, accompanied by shift of ILD_50_ or peak ILD – for the Off-midline-selective ILD function in this panel, as SNR decreased, initially, maximum response strength increased (compare the peak response of the ILD function in the no-noise condition with the one in SNR = 0 dB), then decreased (compare the peak response of the ILD function in SNR = 0 dB condition with the one in SNR = −20 dB). For this ILD function, the peak ILD shifted to ILDs favouring more contralateral locations compared with conditions with higher SNRs (including no-noise condition); (D) shift of ILD_50_ or peak ILD to more lateral locations while response strength remained unchanged – for the Contra-selective ILD function in this panel, as SNR decreased, the ILD_50_ shifted to ILDs favouring more contralateral locations compared with conditions with higher SNRs (including the no-noise condition); (E) change in the shape (category) of the ILD function – the shape of the Off-midline-selective function in this panel changed to Contra-selective; (F) non-systematic changes, i.e. intermittent increase and decrease in response strength as SNR decreased – for instance, note the maximum response of the exemplar ILD function in this panel when SNR decreased from 40 dB to 30 dB and then to 20 dB. No noise: no background noise; S/N: signal-to-noise ratio (in dB); Ipsi: sound pressure level in the ipsilateral ear; Contra: sound pressure level in the contralateral ear.

For 52 response components, after testing at the standard SNR, good spike isolation allowed testing of the effects of increasing level of background noise (decreasing SNR). As shown in Fig.5A, all categories of ILD functions were tested at several SNRs, although due to the time constraint imposed by stability of recording, not all 52 were tested over the same range of SNRs.

Nine of the 52 response components had ILD-insensitive or complex ILD functions and are discussed later. For the remaining 43 response components with monotonic or non-monotonic ILD sensitivity functions, the effect of changing SNR on ILD functions was variable across the response components. Figure5B–F show examples of each of the different patterns of effects by varying SNR observed for the ILD functions.

For some ILD functions, response strength consistently decreased with increasing noise level and this decrease was accompanied by a lateral shift of ILD_50_ (Fig.5B) or Peak ILD compared with the no-noise condition. For another group of ILD functions, the initial effect of increasing noise level was an increase in maximum response strength (Fig.5C; e.g. compare SNR = 0 dB with no-noise condition), followed by a decrease with further increases in noise level (Fig.5C; e.g. compare SNR = −20 dB with SNR = −10 dB). This decrease was also accompanied by a shift in Peak ILD (Fig.5C) or ILD_50_. For a few ILD functions, the only effect was a lateral shift of ILD_50_ with no significant change in maximum response strength (Fig.5D). The other common effects were a change in shape (Fig.5E) and non-systematic changes with changes in SNR (Fig.5F). Figure5E shows an ILD function that in the no-noise condition was Off-midline-selective, but it turned into a Contra-selective ILD function when the level of noise increased, and Fig.5F shows an example of non-systematic changes as the noise level increased above standard.

**Figure 6:**
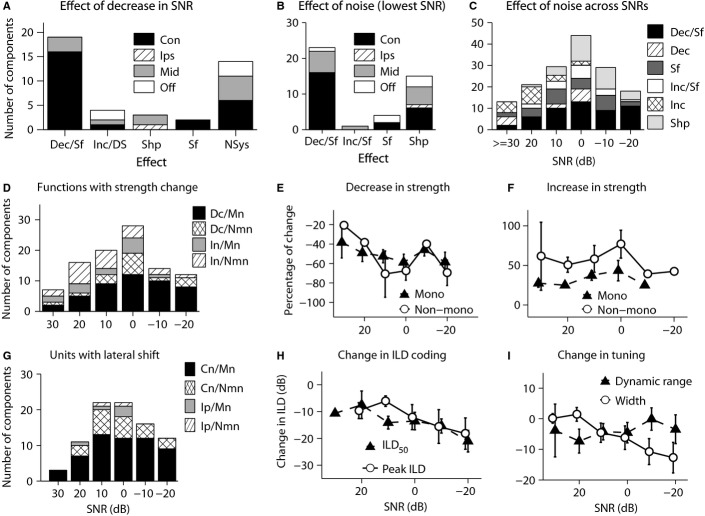
Summary of the effect of decrease in SNR on ILD functions, across all response components (43 response components with monotonic or non-monotonic ILD functions), at the standard ABL. (A) Distribution of pattern of changes in ILD functions as SNR decreased for different ILD categories. (B) Change in ILD function at the lowest SNR vs. ILD function in the no-noise condition, for different ILD categories. (C) Change in ILD function at each SNR compared with the no-noise condition. (D–I) Change in metrics defining ILD functions, i.e. maximum response strength, ILD_50_, peak ILD, 80% dynamic range and width, as a function of SNR, for monotonic (‘Mono’; Contra-selective and Ipsi-selective) and non-monotonic (‘Non-mono’; Midline-selective and Off-midline-selective) ILD functions. (D) Number of ILD functions at each SNR, with significant decrease or increase in maximum response strength, compared with the no-noise condition. (E) Average percentage of significant decrease in maximum response strength, comparing noise with no-noise conditions, as a function of SNR, plotted separately for monotonic (‘Mono’) and non-monotonic (‘Non-mono’) ILD functions. The number of these ILD functions at each SNR is shown in D (‘Dc/Mn’ and ‘Dc/Nmn’, respectively). (F) As for E but showing data for significant increases in maximum response strength. The number of these ILD functions at each SNR is shown in D (‘In/Mn’ and ‘In/Nmn’, respectively). (G) Number of ILD functions tested at different SNRs with significant shift in ILD_50_ or peak ILD, vs. the no-noise condition. (H) Average change in ILD_50_ for monotonic and peak ILD for non-monotonic ILD functions, vs. the no-noise condition, as a function of SNR for ILD functions with significant change in these characteristics. The number of these ILD functions at each SNR is shown in G for monotonic (‘Cn/Mn’ and ‘Ip/Mn’) and non-monotonic (‘Cn/Nmn’ and ‘Ip/Nmn’) ILD functions. (I) Average change in 80% dynamic range (‘Dynamic range’) for monotonic and width for non-monotonic ILD functions as a function of SNR. The number of these ILD functions at each SNR is shown in Fig.5A (‘Monotonic’ and ‘Non-monotonic’). Error bars in E, F, H and I are standard error of means. Con: Contra-selective; Ips: Ipsi-selective; Mid: Midline-selective; Off: Off-midline-selective; Dec: decrease in response strength; Inc: increase in response strength; Sf: shift in ILD_50_ or peak ILD; Shp: change in shape (category) of ILD function; Dec/Sf: decrease in response strength plus lateral shift of ILD_50_ or peak ILD; Inc/DS: increase in response strength followed by decrease in response strength plus lateral shift of ILD_50_ or peak ILD; Inc/Sf: increase in response strength plus lateral shift of ILD_50_ or peak ILD; Nsys: non-systematic; Dc: decrease in response strength; In: increase in response strength; Mn: monotonic ILD functions; Nmn: non-monotonic ILD functions; Cn: contralateral shift; Ip: ipsilateral shift; Mono: monotonic ILD functions; Non-mono: non-monotonic ILD functions.

Figure6A shows the distribution of these patterns of change for the different categories of monotonic (‘Con’ and ‘Ips’ labels) and non-monotonic (‘Mid’ and ‘Off’ labels) ILD functions, for all 43 response components. Most ILD functions (54.7%) eventually exhibited a decrease in maximum response strength accompanied by lateral shift [Fig.6A; decrease in maximum response strength accompanied by lateral shift (‘Dec/Sf’) or increase in maximum response strength then followed by a decrease accompanied by lateral shift (‘Inc/DS’)]. However, a relatively large percentage of functions (33.3%) showed non-systematic changes with increasing noise level (Fig.6A; bars labelled ‘NSys’). With respect to ILD function category, with decreasing SNR, the Contra-selective ILD functions predominantly showed a decrease in response strength accompanied by lateral shift, with the next most common effect being non-systematic changes. The Midline-selective ILD functions also predominantly showed these two effects but the weighting was reversed, with more of this category of function showing non-systematic effects than any other effect. The other two categories of ILD functions were too small in number to draw any firm conclusions with respect to these ILD function categories.

For these 43 response components, the maximal effect of noise within the range of noise levels tested was quantified at the lowest SNR (highest test noise level) and this result is shown in Fig.6B. For most ILD functions (53.4%), the main effect at the highest test noise level was a decrease in response strength accompanied by a lateral shift in the ILD_50_ or peak ILD of the function (left bars labelled ‘Dec/Sf’ in Fig.6B). Many ILD functions (34.8%; Fig.6B; bars labelled ‘Shp’) changed shape (ILD function category). With respect to ILD function category, at the lowest SNR, the Contra-selective ILD functions predominantly showed a decrease in response strength accompanied by lateral shift, with the next most common effect being change in shape (category) of the ILD function. The Midline-selective ILD functions also predominantly showed these two effects, while in contrast to Contra-selective functions, these two effects seemed to be equally likely for this category. The other two categories of ILD functions were too small, and it was not possible to make firm conclusions for these categories. Comparing these results with the results of introducing background noise at the lowest level tested (standard noise) presented in Fig.4F shows that while suppression and facilitation of responses were equally likely at higher SNRs, suppression of the responses became the most dominant effect at lower SNRs.

Effects across all SNRs for the 43 response components are summarized in Fig.6C. Increase in response strength (clear bars and cross-hatched bars, labelled ‘Inc/Sf’ and ‘Inc’) was more prevalent at higher SNRs (lower noise levels), the change in shape (light grey bars, labelled ‘Shp’) was more prevalent at lower SNRs, and decrease in response strength, whether accompanied by lateral shifts (black bars, labelled ‘Dec/Sf’) or not (diagonally hatched bars, labelled ‘Dec’), was found at all SNRs, although all types of changes could be observed at different SNRs, and this suggests that the type of the change in ILD function was not solely dependent on the SNR and varied across populations.

One Complex and eight ILD-insensitive functions were also tested at multiple SNRs. It was noted earlier that nearly all ILD-insensitive functions turned into Contra-selective ILD functions in standard background noise. As SNR decreased, the ILD_50_ of these Contra-selective ILD functions shifted even further towards more contralateral locations compared with the standard noise condition. These functions eventually became non-responsive at very low SNRs. For the only ILD-insensitive function for which standard background noise resulted in suppression of responses at all ILDs, the function remained ILD-insensitive across all SNRs, becoming even more suppressed as the level of noise increased. The only Complex function tested at multiple SNRs exhibited non-systematic changes as SNR decreased.

Finally, we address the issue of the effect of noise on the onset and late response components of Onset-late cells. Eight Onset-late cells with similar ILD function category for onset and late components were tested at a number of SNRs. For five cells, decreasing SNR produced the same effect on onset and late components. Another two cells initially exhibited different effects of background noise for onset and late response components at the standard SNR, i.e. increase of response strength for the onset responses, but change in shape and decrease in response strength for the late responses. When SNR decreased, the first cell showed a change in the category of the ILD function for both onset and late response components, while the second showed non-systematic changes for both response components. For the eighth cell, the effect of decreasing SNR on the ILD functions was different for onset and late response components, with decrease in SNR causing the onset response component’s ILD function to change category, but causing a non-systematic change in ILD function for the late response component. For this cell, the effects of the standard noise on the ILD function of the onset and late response components were also different from each other, i.e. decrease in response strength for onset and increase in response strength for late response component. It was not possible to make this comparison for the Onset-late cells for which the onset and late response components of one cell fell in different categories of ILD functions.

### Quantifying the effect of varying background noise level on ILD sensitivity

We quantified the effect of varying SNR on maximum response strength, putative ILD coding metrics (ILD_50_ for monotonic functions and peak ILD for non-monotonic functions) and tuning characteristics (80% dynamic range for monotonic functions and width for non-monotonic functions) of the ILD functions.

For change in maximum response strength, Fig.6D shows the number of ILD functions that exhibited a significant decrease in maximum response strength at each SNR for monotonic (bars marked ‘Dc/Mn’) and non-monotonic (‘Dc/Nmn’) ILD functions, as well as ILD functions that exhibited a significant increase in maximum response strength for monotonic (‘Inc/Mn’) and non-monotonic (‘Inc/Nmn’) ILD functions. For ILD functions that showed a significant decrease in maximum response strength, Fig.6E shows the mean percentage decrease in maximum response strength compared with the no-noise condition at each SNR for all such ILD functions. Equivalently, Fig.6F shows the mean percentage increase in maximum response strength for ILD functions that exhibited a significant increase in maximum response strength. The mean effect varied somewhat with SNR, especially in the case of the ILD functions exhibiting a decrease. The magnitude of increase in maximum response strength appeared in general to be higher for non-monotonic than for monotonic functions, but there was not a large difference between these groups in terms of decrease in maximum response strength. For each type of change (decrease or increase), there was no significant variation with SNR, for monotonic functions (Kruskal–Wallis test; decrease: *h* = 2.3, d.f. = 5, *P* = 0.8; increase: *h* = 1.7, d.f. = 4, *P* = 0.7) or for non-monotonic functions (Kruskal–Wallis test; decrease: *h* = 4.2, d.f. = 5, *P* = 0.5; increase: *h* = 2.7, d.f. = 5, *P* = 0.7).

For shift in coding features, in Fig.6G, the bars marked ‘Cn/Mn’ and ‘Cn/Nmn’ show the number of ILD functions that in background noise exhibited a significant shift in ILD_50_ and peak ILD, respectively, to more contralateral locations compared with the no-noise condition, while ‘Ip/Mn’ and ‘Ip/Nmn’ show the same shift to more ipsilateral locations. The majority of the ILD functions shifted towards more contralateral locations compared with the no-noise condition. Figure6H shows the mean of the distributions of change in ILD_50_ and peak ILD across SNRs. Again, there was no significant variation with SNR for monotonic (Kruskal–Wallis test; *h* = 8.8, d.f. = 5, *P* = 0.1) or non-monotonic (Kruskal–Wallis test; *h* = 7.3, d.f. = 5, *P* = 0.1) ILD functions.

Finally, Fig.6I shows the mean change in tuning characteristics caused by noise as indexed in the 80% dynamic range for monotonic functions and in width for non-monotonic functions. These values were computed for all monotonic and non-monotonic ILD functions, regardless of the change in response strength. The numbers of functions contributing to these means were reported earlier in Fig.5A (bars marked as ‘Monotonic’ and ‘Non-monotonic’ at each SNR). The change in these characteristics at each SNR varied across ILD functions and there was no significant variation with SNR for 80% dynamic range (Kruskal–Wallis test; *h* = 2.2, d.f. = 5, *P* = 0.8) or for width (Kruskal–Wallis test; *h* = 9.2, d.f. = 5, *P* = 0.1). In summary, the magnitude of change in characteristics of the ILD functions was not solely dependent on SNR and varied across populations.

**Figure 7:**
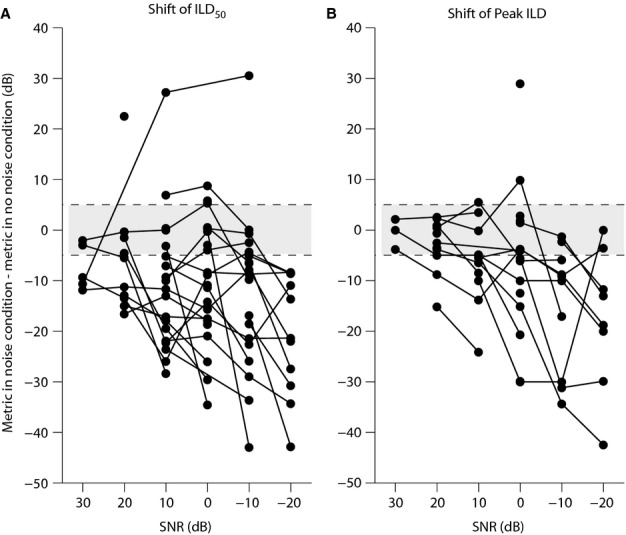
Effect of varying SNR on putative coding measures of space at the standard ABL. (A) Shift in ILD_50_ of monotonic ILD functions. (B) Shift in peak ILD of non-monotonic ILD functions. Shift is measured as the metric, i.e. ILD_50_ or peak ILD, at each noise condition (i.e. each SNR) minus the same metric in the no-noise condition (i.e. negative values represent shift towards more contralateral ILDs). The shaded area represents the significance margin of the shift of ILD_50_ or peak ILD, i.e. between −5 and 5 dB. The shift in these metrics is significant if it is outside this margin.

Considering the putative role of ILD_50_ and peak ILD in coding of space, to examine the effect of noise on these metrics in greater detail, we plotted the change in ILD_50_ and peak ILD with respect to the no-noise condition vs. SNR, individually for all the ILD functions tested (Fig.7). In this figure, in addition to the significant shifts considered in Fig.6H, the shifts that were not significant (the shifts between −5 and 5 dB) were also shown. Although background noise significantly affected these metrics for the majority of ILD functions at most SNRs (points falling out of the shaded area at each SNR in Fig.7), the ILD_50_ or peak ILD of a number of ILD functions was not significantly affected by background noise (points for each ILD function falling in the shaded area at each SNR), even at relatively high levels of noise. Also, it seems that peak ILD of the non-monotonic functions is more resistant to noise, as at lower levels of noise (SNR < 10 dB) the peak ILD of a larger percentage of ILD functions seems to show non-significant shifts (points falling in the shaded area at each SNR in Fig.7B), compared with the ILD_50_ of the monotonic functions (points falling in the shaded area at each SNR in Fig.7A). However, this conclusion must be heavily qualified by the small number of ILD functions available for analysis for both monotonic and non-monotonic ILD functions.

### Excitatory and inhibitory inputs and correlation with the effect of background noise

**Figure 8:**
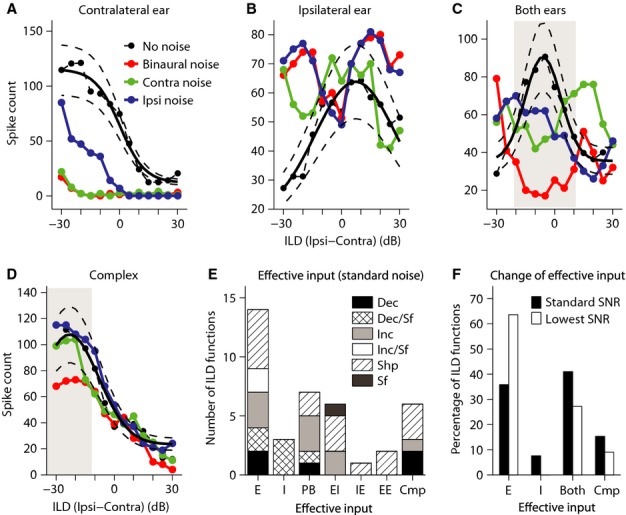
Sources of effects of binaural background noise on ILD sensitivity functions. The panels present examples of ILD functions for which the effect of binaural background noise was mainly due to the effect of noise on (A) input from the contralateral ear (note similarity between ILD functions marked ‘Contra noise’ and ‘Binaural noise’) or (B) input from the ipsilateral ear (note similarity between ILD functions marked as ‘Ipsi noise’ and ‘Binaural noise’), or due to (C) the combination of effects on inputs from both ears [the exemplar ILD function here was recorded from a predominantly binaural (PB) cell], and changes in this ILD function in the ‘Binaural noise’ condition could be explained with respect to binaural facilitation in this type of cell; e.g. suppression of the responses in the shaded area for the ILD function marked as ‘Binaural noise’ seemed to be the combination of suppression of responses in the ‘Contra noise’ and ‘Ipsi noise’ conditions. (D) For some ILD functions, the effect of binaural noise seemed to be the result of a complex effect on inputs. For the exemplar ILD function here, while responses to ILDs in the shaded area were suppressed in the ‘Binaural noise’ condition, neither ILD function in the ‘Contra noise’ or ‘Ipsi noise’ conditions exhibited significant suppression at these ILDs. So, it is not possible to explain the effect of binaural noise based on the effect solely on one monaural input or a combination of these effects. The thick solid line shows best fit to the ILD responses for each response component, and dashed lines represent the margin of 20% of the response at different ILDs. Change in response strength in the noise condition compared with the no-noise condition was considered non-random if the response strength fell outside this margin. (E) Distribution of type of effective input for different effects of binaural background noise at the standard SNR for 39 ILD functions that were tested in monaural and binaural background noise conditions. (F) Comparison between the distributions of type of effective input that was adequate to explain the effect of binaural masking at the standard SNR and at the lowest SNR tested (for 16 ILD functions tested at different SNRs). No noise: no background noise; Binaural noise: binaural noise; Contra noise: monaural noise in contralateral ear; Ipsi noise: monaural noise in ipsilateral ear; E: excitatory; I: inhibitory; PB: predominately binaural; Cmp: complex mixture of effects on monaural inputs. Dec: decrease in response strength; Inc: increase in response strength; Sf: shift in ILD_50_ or peak ILD; Shp: change in shape (category) of the ILD function; Dec/Sf: decrease in response strength plus lateral shift of ILD_50_ or peak ILD; Inc/Sf: increase in response strength plus lateral shift of ILD_50_ or peak ILD; Ipsi: sound pressure level in ipsilateral ear; Contra: sound pressure level in contralateral ear.

To attempt to elucidate the mechanisms underlying the effect of binaural noise on ILD sensitivity and to interpret the changes in terms of likely effects on excitation and inhibition, we examined the effects of presenting background noise to each ear monaurally at the standard noise level on ILD sensitivity at the standard ABL. We were able to record responses to ILDs under the different noise laterality conditions at the standard noise level (standard SNR) for 39 ILD functions, and Fig.8A–D show examples of the patterns of effects observed. Only noise-induced changes in the ILD function by more than 20% of the response in the no-noise condition at each ILD were considered to be non-random. This margin is shown using dashed lines in these figures.

Figure8A shows an example where the effects of binaural noise were replicated by noise presented only to the contralateral ear (compare the ILD functions marked as ‘Contra noise’ and ‘Binaural noise’), suggesting that the effective input to this response component emanated via the contralateral ear, and suppression of only this input could account for the effect of binaural noise. The alternative case is illustrated in the non-monotonic function in Fig.8B where noise presented only to the ipsilateral ear evoked the same effect as noise presented binaurally (compare the ILD functions marked as ‘Ipsi noise’ and ‘Binaural noise’), suggesting that, here, the effective input was from the ipsilateral ear, and suppression of only this input was sufficient to account for the effect of binaural noise. In Fig.8C, introduction of noise in either the contralateral or the ipsilateral ear resulted in suppression of responses at ILDs ranging from approximately −20 to 10 dB (shaded area), although response suppression was stronger with binaural noise, suggesting that the effect of binaural background noise was the result of the combination of the effects of noise on both ears. The cell from which this response component was recorded was an EE/F cell, also referred to as predominately binaural (PB) (Goldberg & Brown, 1969), and received excitatory inputs from both ears. In this type of cell, binaural stimulation results in a strong response that is larger than the sum of the monaural responses, namely facilitation. It seems that for this response component, when monaural responses were suppressed with binaural background noise, the monaural excitation received from the two ears was not strong enough to induce facilitation as opposed to the no-noise condition, inducing strong suppression in the responses.

For ILD functions like that in Fig.8D, the effect of binaural noise could not be accounted for by changes observed when noise was introduced monaurally to either ear or by a combination of these changes. Rather, a complex mixture of effects exerted via both ears appeared responsible for the effect of binaural noise. For example, for the ILD function in Fig.8D, when noise was introduced monaurally in either ear (ILD functions marked as ‘Contra’ and ‘Ipsi’), any change in the ILD function fell within the 20% response variability margin (represented by the dotted lines). However, when noise was presented binaurally, there was a strong suppression in response strength at ILDs ranging approximately from −30 to −20 dB (shaded area), and responses fell below the response variability boundary.

Using these definitions, we found the effective input for each ILD function, namely the ear via which monaural noise could account for the effects of binaural noise on the ILD function at the standard SNR, and these results are presented in Table1.

**Table 1:** Distribution of the effective ear(s) for each category of ILD functions at the standard SNR

	Effective ear			
	Contra ear	Ipsi ear	Combination	Complex effect
Contra-selective	5	3	4	4
Ipsi-selective	0	0	2	0
Midline-selective	1	0	4	1
Off-midline-selective	3	0	1	0
ILD-insensitive	3	0	3	0
Complex	1	1	2	1

Contra ear: effect on input from contralateral ear; Ipsi ear: effect on input from ipsilateral ear; Combination: combination of effects on inputs from both ears; Complex effect: complex mix of effects on inputs from the ears.

For Contra-selective ILD functions, the effect of binaural noise seemed to be explicable by any of the effects described above (i.e. the effect of noise on either ear, a combination of effects via the two ears, or a complex mixture of effects via the two ears; first row of Table1), and for the Midline-selective functions by the combination of the effects via the two ears (third column of the third row). For the rest of the categories the numbers were too small to make a firm conclusion.

As described in the Materials and methods, we identified the binaural input characteristics of the response components in the no-noise condition, and these characteristics for the 39 response components examined are presented in Table2. The majority of the cells (43.5%) were EI, and a second large group of cells (28.2%) were predominantly binaural (PB), a category that comprised EO/F, OO/F and EE/F cells (see Materials and methods for definition). These results and binaural input characteristics of each category of ILD functions were in accordance with those reported in IC of cat (Geisler & Rhode, 1969; Moore & Irvine, 1981; Irvine, 1986; Caird & Klinke, 1987; Irvine & Gago, 1990; Irvine *et al*., 1996) and gerbil (Semple & Kitzes, 1987), for which such data have been reported.

**Table 2:** Distribution of binaural type for each category of ILD function

	Binaural characteristics							
	EI	IE	EE	OE/F+I	EO/F	OO/F	EE/F	EO/mon
Contra-selective	14	0	0	0	0	0	1	1
Ipsi-selective	0	1	0	0	0	0	1	0
Midline-selective	0	0	0	0	2	0	3	1
Off-midline-selective	1	0	1	0	0	1	0	1
ILD-insensitive	0	0	4	0	0	0	2	0
Complex ILD function	2	0	0	1	1	0	0	1

E, excitatory; I, inhibitory; F, facilitatory; O, occluded; mon, monaural.

We used the results of our analysis of identifying the effective ear presented earlier (Table1) along with these binaural characteristics (Table2) to make conclusions about the excitatory and inhibitory inputs that were affected by background noise and that mediated the effect of binaural background noise on each ILD function at the standard SNR.

These results separated for different effects of binaural masking on the ILD functions at the standard SNR are shown in Fig.8E. Where the effect of binaural noise could be explained solely by effects on one ear or the other, the figure separates effects of binaural noise according to whether the effect could be explained solely by effects on a monaural excitatory input (‘E’; leftmost bars) or solely by effects on a monaural inhibitory input (‘I’; second bar). Where the effects of binaural noise were explained by effects on both ears, the figure plots separately the effects on each of the different types of binaural interaction (i.e. ‘PB’, ‘EI’, ‘IE’ and ‘EE’). Of 39 ILD functions, at the standard SNR, the effect of binaural masking for the majority of the ILD functions (41%) was due to a combination of inputs received from both ears (‘PB’, ‘EI’, ‘IE’ and ‘EE’ in Fig.8E). For a large percentage of ILD functions (35.8%), regardless of the exact effect induced by noise (decrease, increase, etc.), the effect of binaural background noise seemed to be due to an effect only on excitatory input (‘E’; leftmost bar in Fig.8E), while for a small percentage of ILD functions (7.6%), the effect of binaural background noise seemed to be due to the effect only on inhibitory input (‘I’; second bar in Fig.8E). For 15.3% of the ILD functions, it was not possible to explain the result of binaural masking based on the effect on individual ears (extreme right bar in Fig.8E). Figure8E shows that various changes observed as a result of introducing binaural background noise, i.e. increase (‘Inc’ and ‘Inc/Sf’) or decrease (‘Dec’ and ‘Dec/Sf’) in maximum response strength, or change in shape (‘Shp’), could be either due to the effect of noise solely on the excitatory input or due to the combination of the effects on inputs from both ears, as well as a complex mixture of effects on the inputs from the ears. The only effect of noise that was explained by the effect solely on the inhibitory input was decrease in the maximum response strength accompanied by lateral shift of the ILD function (cross-hatched bar labelled ‘I’). Decrease in maximum response strength (‘Dec’ and ‘Dec/Sf’) was mostly due the effect of noise on the excitatory input (36.3%) or inhibitory input (27.2%) as opposed to the combination of effects on the inputs from the two ears (18.1%), while for the increase in maximum response strength (‘Inc’ and ‘Inc/Sf’) the effect could be due either to the change in excitatory input (45.5%) or to the combination of changes in the two ears (54.5%). Change in shape (‘Shp’) for the majority of the ILD functions was explained by the combination of changes on the inputs from both ears (50%), although it could also be due to the change in monaural excitatory input (31.2%).

With respect to the category of the ILD functions, for Contra-selective ILD functions, excitatory input (31.2%) followed by interaction between excitatory and inhibitory inputs (25%) (EI cells) predominantly explained the effect of binaural background noise, as well as a complex mixture of the effect on the inputs from the two ears (25%). For Midline-selective ILD functions, it seemed that change in inputs from both ears and the interaction between these inputs for the predominately binaural (PB) cells explained the effect of binaural background noise for the majority of cells (66.6%). For the rest of the categories, the numbers were too small to make a firm conclusion.

Again, we examined the issue of how noise affected the different temporal components from the same Onset-late cell. For three Onset-late cells that had the same category of ILD functions for onset and late response components, the effective ears for the two response components were different. For these three cells, the effective ear was complex (one case) or excitatory (two cases) for the onset response components, while for their corresponding late response components, it was inhibitory, EE or complex, respectively. For three other Onset-late cells that had the same category of ILD functions for onset and late response components, the effective ears for the two components were the same.

### Effect of varying SNR on the interaction between inputs from the ears

In the last section, we reported results obtained by analysing the interaction between the excitatory and inhibitory inputs only at the standard SNR. For 16 response components, we decreased the SNR to investigate the effect of noise level on the effective input when tonal stimuli were presented at the standard ABL.

For 11 of these ILD functions (68.7%), the effective input remained the same across different levels of noise introduced. For the remaining five ILD functions, the effective input at the lowest SNR tested was different from the effective input at the standard SNR. For example, for one ILD function, the effect of background noise on inhibitory input was accountable for the effect of binaural background noise on the ILD function at the standard SNR, while when SNR was at the lowest level tested, the effect of binaural background noise was explicable by the effect of background noise on excitatory input. For the other four of these five ILD functions, a similar change was observed: for two ILD functions, the effective input changed from being a combination of the inputs from both ears into excitatory input, for one ILD function, from inhibitory input to a combination of inputs from both ears, and for the last ILD function, from excitatory input into a complex mixture of the effects on monaural inputs. Figure8F shows a comparison between the effective ear at the lowest SNR tested and at the standard SNR for all these 16 ILD functions. It can be seen that at lower SNRs, for a larger percentage of ILD functions (63.6%) the effect of introducing binaural background noise could be explained solely by the effect on excitatory input, compared with the higher standard SNR (35.8%) (Fig.8F; leftmost bars). This is while, still for some ILD functions, at the lowest SNRs, the effective ear was either a combination of the effects on the two ears (clear bar labelled ‘Both’ in Fig.8F) or a complex mixture of the effects on the monaural inputs (clear bar labelled ‘Cmp’ in Fig.8F). As can be seen in Fig.8F, for none of the ILD functions was inhibitory input per se enough to account for changes observed as a result of introducing binaural background noise at the lowest SNRs tested. Finally, because the number of cells in each category of ILD functions was small, we were not able to arrive at reliable conclusions regarding the effective ear for different categories of ILD functions and also for various effects of binaural masking on the ILD function, and the results are not reported here.

### Effect of ABL on category of ILD functions and the effect of masking

The results described thus far were obtained with the CF stimulus presented at the standard ABL. As ILD sensitivity can vary with ABL (Semple & Kitzes, 1987; Irvine & Gago, 1990), when recording conditions permitted, we recorded responses at other ABLs as well. For 26 response components, we were able to examine the effect of noise masking on ILD functions at a number of ABLs higher than the standard ABL.

**Figure 9:**
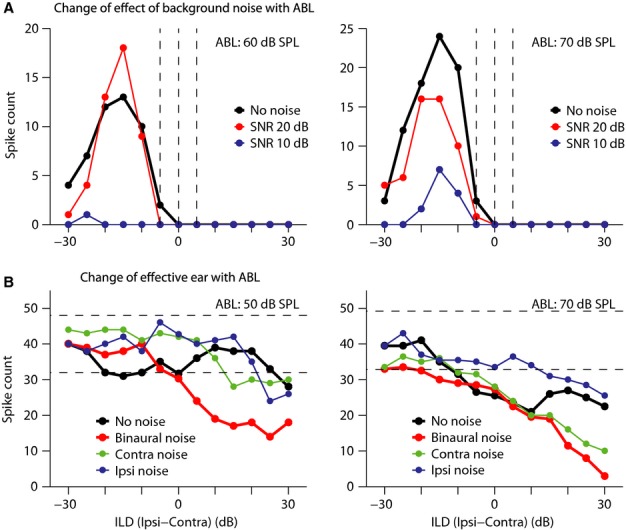
Effect of the change in ABL on the effect of background noise and effective input. (A) Change in the effect of binaural background noise with change in ABL. The effect of noise on the Off-midline-selective ILD function of the exemplar response component in this figure at ABL 60 dB SPL (standard ABL) is an increase in response strength followed by decrease (left panel), while at ABL 70 dB SPL, it is a decrease in response strength (right panel). (B) Change in the effective monaural input with ABL. For this example, the effect of binaural noise on the ILD function at ABL 50 dB SPL (standard ABL) can be explained by a combination of effects on inputs from both ears, while it can be explained based on the input from the contralateral ear at ABL 70 dB SPL (right panel). Note that the category of the ILD function for both examples does not change with change in ABL (as was the case for the majority of the ILD functions tested at multiple ABLs in this study). No noise: no background noise; Binaural noise: binaural background noise; Contra noise: monaural background noise in the contralateral ear; Ipsi noise: monaural background noise in the ipsilateral ear; Ipsi: sound pressure level in the ipsilateral ear; Contra: sound pressure level in the contralateral ear.

For most response components (20/26; 76.9%), when ABL was increased above the standard ABL, the category of the ILD functions remained unchanged across ABL levels. For example, in Fig.9A the category of the ILD function in the no-noise condition (black traces) was Off-midline-selective at the standard ABL of 60 dB SPL (left panel) and remained unchanged when ABL increased to 70 dB SPL (right panel). Although the category of the ILD function in Fig.9A did not change when ABL increased, this function was the only ILD function out of these 20 ILD functions for which increasing ABL changed the effect of binaural background noise. For this function, as can be seen in Fig.9A, at an ABL of 60 dB SPL, when binaural noise was presented, there was an initial increase in the response strength at SNR 20 dB followed by a decrease in response strength at the lower SNR of 10 dB (Fig.9A, left panel), whereas at an ABL of 70 dB SPL there was only a decrease in the response strength across all SNRs (Fig.9A, right panel).

For the remaining six ILD functions (23%) that were tested at multiple ABLs, an increase in ABL changed the ILD function category. For two ILD functions, category of the ILD function changed from Off-midline-selective to Complex, and for the other four ILD functions, the category changed from Contra-selective to ILD-insensitive, from Contra-selective to Midline-selective, from Complex to Midline-selective, and from Complex to Ipsi-selective.

Finally, we assessed the effect of ABL on the interaction between inhibitory and excitatory inputs that formed the ILD functions of 11 response components, by examining the effect of monaural noise at multiple levels of ABL. When ABL was raised above the standard ABL, for nearly all response components (90.9%) the input that appeared to be sufficient to explain the effect of binaural masking remained the same. Only for one ILD-insensitive function (black traces in Fig.9B), while introducing background noise at both the standard ABL (50 dB SPL) and at a higher ABL of 70 dB SPL resulted in a change of the category of the ILD function to Contra-selective (red traces in left and right panels of Fig.9B, respectively), the ear accountable for the effect of binaural noise seemed to differ for ABL 50 dB SPL and ABL 70 dB SPL. The change in the responses with noise at the standard ABL (50 dB SPL) seemed to be due to the combination of effects on inputs from both ears. As can be seen in the left panel of Fig.9B, when noise was introduced in either the contralateral or the ipsilateral ear there was a decrease in responses at more positive ILDs, and the decrease in responses in binaural noise appeared to be probably a combination of the observed decrease in the monaural cases. At the higher ABL of 70 dB SPL, the result clearly seemed to be due to effects on input from the contralateral ear (compare the ILD functions marked as ‘Contra noise’ and ‘Binaural noise’ in the right panel of Fig.9B).

## Discussion

We found that background noise had heterogeneous effects on ILD coding by IC neurons and on inputs emanating from each of the two ears to shape that ILD sensitivity. SNR was an important factor influencing the effect of binaural background noise on the ILD functions, e.g. suppression or facilitation of responses, and the excitatory or inhibitory inputs that were probably affected. This will be discussed in detail in the following sections. Also, we found that onset and late response components of the same cell could show different changes with noise, even when they showed the same form of ILD coding. This suggests that the mechanisms shaping ILD sensitivity of the two components may be independent, in keeping with the known differences between the two components for other metrics such as sensitivity at CF, response rate-level function and ILD sensitivity (Hind *et al*., 1963; Geisler & Rhode, 1969; Moore & Irvine, 1981; Irvine, 1986; Irvine & Gago, 1990; Finlayson, 1999), and the fact that the interaction between monaural inputs to an IC cell can be time-dependent (Zhang & Kelly, 2009, 2010). However, as the functional significance of different response patterns in the IC is still not well known (Zhang & Kelly, 2010), and particularly it is unknown how the different components contribute to spatial localization, it is not trivial to discuss the effect of these differences on spatial coding.

### Comparison with previous studies

To our knowledge, this is the first study on the effects of continuous background noise on spatial coding in the central nucleus of the IC. Studies of noise masking effects on spatial coding have been conducted in auditory cortex (Brugge *et al*., 1998; Furukawa & Middlebrooks, 2001) and in SC (Martin *et al*., 2010). Given the obligatory role of the central nucleus of IC in flow of information to both these structures (Irvine, 1986; Pickles, 1988; García Del Caño *et al*., 2006; Slee & Young, 2013), it will be instructive as to whether the effects described in those structures parallel those in the IC or reveal novel emergent effects. However, note that we cannot segregate the IC recordings into the different IC sub-divisions and this impacts on our ability to unequivocally correlate the effects observed in our IC recordings with effects seen in auditory cortex or SC, which get differential projections from different IC sub-divisions.

In cat A1, Brugge *et al*. (1998) found that the effect of noise was mainly to cause a reduction in response strength and shrinkage of the spatial receptive field. However, Martin *et al*. (2010) found that response strength of SC cells of rats mostly did not change in background noise (78.3 and 65% for two different types of receptive fields), while it increased for a small percentage (8.7 and 15%) and decreased for another small percentage (13 and 20%) of cells. Furukawa & Middlebrooks (2001) reported that nearly two-thirds of A2 neurons in cats exhibited response suppression in background noise, but at lower noise levels, one-third of the neurons showed enhancement of responses. Our results suggest that although these differences may reflect differences in processing in different auditory areas, they may also be due to the difference in the noise levels used. We found that at the standard SNR, i.e. the lowest noise level tested, noise caused enhancement (34.8% of ILD functions) as well as suppression (30.3% of the ILD functions), while at the lowest SNR (the highest noise level tested), noise mainly induced suppression of the maximum response strength (55.8% of the ILD functions). A very small number of cells were unaffected by noise (7.4%) within the range of test noise levels (higher levels may have had an effect). In our study, the effects of noise at higher SNRs were more consistent with those observed by Martin *et al*. (2010) in SC, and, in lower SNRs, more similar to those found by Brugge *et al*. (1998). What we found in IC was consistent with Furukawa & Middlebrooks’ (2001) study in A2 that reported cases of facilitation in the responses when SNR was high and mostly suppression of responses when SNR was low.

In addition to changes in response strength, binaural and monaural background noise also caused shifts in the ILD sensitivity functions. It has been shown that ILD sensitivity functions recorded to a probe signal in IC could shift towards the mean ILD of a noise stimulus preceding the probe signal (Dahmen *et al*., 2010). In our study, this cannot be a contributing factor when testing with binaural noise, as noise was always set to be equal in both ears (i.e. ILD = 0 dB). When background noise was presented monaurally to the contralateral ear, for some ILD functions there was a shift of the ILD function to more contralateral locations (compared with the no-noise condition), similar to what has been reported for IC neurons by Dahmen *et al*. (2010). However, for these ILD functions, when monaural noise was presented to the ipsilateral ear, we did not observe a shift to ipsilateral locations, except for two ILD functions. Thus, it seems the shift of ILD functions with monaural or binaural noise is probably due to a mechanism other than adaptation to ILD of background noise.

To quantify these effects of noise on the ILD functions, we used a curve fitting method (Materials and methods) with functions selected to allow best comparison of changes in ILD functions by the objective measurement of metrics from fits to the ILD functions. We do not mean to imply a physiological meaning to the specific fitted function in terms of the physiology underpinning the ILD functions.

### Underlying events causing changes in ILD functions in binaural background noise

It has been speculated that the effect of noise on sound localization is exerted through changes in the balance of inhibitory and excitatory inputs to cells (Finlayson & Adam, 1997; Finlayson, 1999; Furukawa & Middlebrooks, 2001). By comparing the effects of monaural and binaural noise on ILD encoding and then evaluating these effects in the context of presumed dominant monaural excitatory or inhibitory inputs to the IC cells, we found that at low background noise levels, the effects of binaural noise appeared to be the result of either a combination of changes in both monaural inputs (41%) or due solely to a change in the presumed excitatory inputs (35.8%), and as background noise level increased, effects on the presumed excitatory inputs became the dominant effect to account for the effect of binaural background noise (63.6%).

Differential effects on contralateral and ipsilateral inputs in our study cannot be due to differences in effects at the cochlea, as the binaural noise was the same in the two ears and would have the same masking effect at both cochleas (Costalupes *et al*., 1984; Gibson *et al*., 1985). Therefore, monaural-specific changes induced by binaural background noise must be due to some centrally occurring difference in the way differential inputs from the two ears are affected. As we did not directly record inhibitory and excitatory events and cannot know the origin of the inhibitory and excitatory events shaping the ILD sensitivity of IC neurons, speculations on these central changes must be couched in general terms. Furthermore, in interpreting the effects of monaural noise, we assumed a predominant excitatory or inhibitory input derived from the test ear, whereas intracellular recordings have shown both excitatory and inhibitory postsynaptic current to monaural stimulation of either ear (Ono & Oliver, 2014), and Xiong *et al*. (2013) have shown a role for bilateral inhibition in ILD processing in IC. Thus we take a cautious approach to interpretation of the underlying changes in noise effects on ILD coding in IC.

The IC receives a complex mix of excitatory and inhibitory inputs from several brain structures (Burger & Pollak, 2001; Li *et al*., 2010), mainly contralateral lateral superior olive (LSO), contralateral and ipsilateral dorsal nucleus of the lateral lemniscus (DNLL) and contralateral IC (Li & Kelly, 1992; Faingold *et al*., 1993; González-Hernández *et al*., 1996; Burger & Pollak, 2001; Loftus *et al*., 2004; Li *et al*., 2010; Pollak, 2012). LSO provides direct excitatory inputs, while DNLL provides predominant inhibitory inputs to the IC (Burger & Pollak, 2001). Background noise in our study may have caused an adaptation of excitation coming from LSO or inhibition coming from DNLL. Adaptation results in facilitation and suppression of responses in LSO (Finlayson & Adam, 1997) and IC (Finlayson, 1999), although there has been no study of such effects in DNLL, and this must remain speculative.

In LSO of gerbils and IC of bats, Magnusson *et al*. (2008) and Park & Pollak (1993, 1994), respectively, found that GABA plays a role in lateral shift of the monotonic ILD functions, like the noise-induced shift we observed. Magnusson *et al*. (2008) proposed that the GABA-induced change in the ILD functions plays a role in adaptation to the acoustic environment. A similar mechanism and functional role could underlie our observation of a noise-induced shift of the monotonic ILD sensitivity functions in IC mostly to ILDs generated from more contralateral locations compared with the no-noise condition. Also, the inhibitory input from ipsilateral DNLL to IC (Pollak, 2012) is thought to shape non-monotonicity of rate-level functions (Pollak, 2012), and background noise effects on this input may be responsible for the change in shape of the ILD function of some EI cells. Finally, the noise effects we observed could occur through noise-induced activation of surround inhibition (Rajan, 2001), which has been shown to affect the responses of IC neurons (Xie *et al*., 2005).

### Noise-induced change in metrics of ILD functions and relevance to psychophysics

Background noise caused a shift in metrics that could potentially be the neural read-out code for auditory spatial location, namely peak ILD or ILD_50_. ILD is highly variable with frequency and location in rats (Koka *et al*., 2008), so it is not trivial to translate the shift we observed in these metrics directly to location in auditory space or correlate this with performance in sound localization in humans. However, one prediction of the noise-induced shift of ILD functions to ILDs favouring more contralateral locations is that, in noise, there should be an apparent shift in perceived sound location to more lateral locations. Support for this proposition is found in the data of Good & Gilkey (1996), showing the effects of noise on sound localization by humans. They showed that as noise increased, sounds to the left were perceived as being further to the left, and sounds to the right were perceived as being further to the right, consistent with our prediction. This pattern was disrupted only at the highest noise level (−10 dB SNR) when sounds showed a bias to be localized further to the right than they really were, regardless of whether they originated from left or right.

Despite the changes we generally observed for ILD sensitivity functions as a result of background noise, Good & Gilkey (1996) and Good *et al*. (1997) showed that humans maintain their sound localization capability to a large extent, as long as the sound was clearly audible (i.e. SNR ∼10 dB). Some compensation for the effect of background noise on sound localization may start even at the level of cochlea through feedback from the olivocochlear efferent system (May *et al*., 2004; Andéol *et al*., 2011; Reiss *et al*., 2011). Furthermore, the changes observed in ILD sensitivity in our study with background noise may not directly map to poor sound localization. One potential contributor to cochlear implantees’ poor sound localization ability in noise is that they are unable to use cues other than ILD, such as interaural time differences or spectral cues, and rely heavily on ILD for sound localization (Van Hoesel & Tyler, 2003; Seeber & Fastl, 2008). This suggests that these other monaural and binaural cues could help in sound localization in noise while ILD sensitivity was disrupted by background noise. Some other coding characteristics of higher auditory area cells coding for space also may remain intact when background noise is present, and this may help in sound localization in noise. For example, Brugge *et al*. (1998) proposed that the gradient of the receptive field is a code for auditory space, and showed that the slope and position of this gradient remained constant across noise conditions. Finally, in our study, we found a small number of ILD functions that were unaffected by noise. This is similar to the zebra finch auditory cortex noise-invariant neurons (Moore *et al*., 2013) that showed similar responses to bird songs in the absence and presence of background noise. We also found moderate stability of the ILD_50_ or peak ILD of some ILD functions, when background noise was introduced. The small sample size precludes any claim that this relative stability of ILD functions is a common mechanism in allowing IC cells to maintain coding of space in the presence of noise. However, it does indicate that some IC cells show relative stability of putative coding mechanisms for spatial location in the presence of noise, analogous to the effects reported in SC (Martin *et al*., 2010).

## Conclusion

Our findings suggest that many effects of background noise on spatial encoding in upstream brain areas, such as A1, A2 and SC (Brugge *et al*., 1998; Furukawa & Middlebrooks, 2001; Martin *et al*., 2010), may reflect effects at the auditory midbrain or other downstream structures. It is unlikely that the midbrain effects reflect effects relayed from cortex because of the fast time scale (e.g. changes in the IC onset response, occurring within 5–10 ms from stimulus onset, when afferent responses are only just starting to occur in auditory cortex), and because the barbiturate anaesthetic we used significantly suppresses even primary auditory cortical responses (see Discussion in Rajan *et al*., 2013).

## Abbreviations

ABL: average binaural level
binaural noise: binaural background noise
CF: characteristic frequency
Cmp: Complex
Con: Contra-selective
Contra: sound pressure level in the contralateral ear
contra noise: monaural background noise in the contralateral ear
Dec/Sf: decrease in response strength accompanied by lateral shift of ILD_50_ or peak ILD
Dec: decrease in response strength
E: excitatory
I: inhibitory
IC: inferior colliculus
ILD: Interaural level difference
Inc/DS: increase in response strength followed by decrease in response strength
Inc/Sf: increase in response strength accompanied by lateral shift of ILD_50_ or peak ILD
Inc: increase in response strength
Ins: ILD-insensitive
Ips: Ipsi-selective
Ipsi noise: monaural background noise in the ipsilateral ear
Ipsi: sound pressure level in the ipsilateral ear
Mid: Midline-selective
Nsys: non-systematic
no noise: no background noise
Off: Off-midline-selective
PB: predominately binaural
SC: Superior Colliculus
Sf: shift in ILD_50_ or peak ILD
Shp: change in the shape, namely category, of the ILD function
